# Evaluation of Cortactin and *HS1* Genes Expression: New Players in Adult B-Cell Acute Lymphoblastic leukemia

**DOI:** 10.31557/APJCP.2021.22.3.767

**Published:** 2021-03

**Authors:** Salah Aref, Mohamed Al Agdar, Ahmed Ramez, Tarek Abou Zeid, Mohamed Sabry, Nada Khaled

**Affiliations:** 1 *Hematology Unit, Department of Clinical Pathology, Faculty of Medicine, Mansoura University, Egypt. *; 2 *Medical Oncology Unit, Mansoura University Oncology Center (MUOC) Mansoura University, Egypt. *; 3 *Hematology Unit, Mansoura University Oncology Center (MUOC), Mansoura University, Egypt. *

**Keywords:** Cortactin, HS1, ALL, outcome

## Abstract

**Objectives:**

This study aimed to assess the prognostic value of cortactin and *HS1* genes expression in adult B-cell acute lymphoblastic leukemia.

**Methods:**

The study included a cohort of 74 adult B-ALL patients and 76 controls. Cortactin and *HS1* genes expression were quantified by real time PCR.

**Results:**

The expression of cortactin and HS1 were significantly higher in B-ALL patients at diagnosis as compared to post induction levels (P<0.001) as well as normal controls (P<0.001 for all). Cox regression analysis revealed that B-ALL patients with high Cortactin expression as well as high HS1 expression had significant high risk of relapse (P=0.005; Odds ratio (OR)= 1.428, CI= [1.175-1.783]; and P=0.003; OR= 1.078, CI= [1.025-1.134]; respectively) and higher probability of deaths (P= 0.041; OR=1.092, CI =[1.002-1.04]; and P=0.005; OR=1.071, CI=[1.013-1,041]; respectively). Survival analysis revealed that B-ALL patients with high cortactin and high *HS1* expression had significantly shorter OS and increased frequency of relapse as compared to those with lower expression levels (P <0.01 for all).

**Conclusion:**

High cortactin and *HS1* genes expression at diagnosis denote bad clinical outcome in B-ALL patients. Assessment of correction expression at B-ALL diagnosis could be considered as risk biomarker at diagnosis.

## Introduction

Acute lymphoblastic leukemia (ALL) is an abnormal proliferation and differentiation of a clonal population of lymphoid cells within bone marrow and blood stream. About 80% of ALL occurs in children with a median age of 16 at diagnosis. In adults, ALL is less common than acute myeloid leukemia (AML) but when occurs, it represents a devastating disease (Terwilliger et al., 2017). ALL follows a bimodal distribution; the first peak occurs in patients around 5 years of age followed by a second peak at around 50 years of age (Paul et al., 2016). In ALL the leukemic cells arise from either B-cell (B-ALL) or T-cell (T-ALL) progenitors. B-ALL accounts for about 80% of ALL cases and occur at any age, however it mainly affects children. (Chiarini et al., 2016). Although the new treatment modalities resulted in an increased overall survival (OS) up to 80%, there are still a percentage of patients that develop treatment resistance and relapse (Bhojwani and Pui 2013). Bone marrow (BM) relapse most common than extramedullary infiltration such as central nervous system (CNS); with a generally worse survival (Malempati et al., 2007). Several studies have demonstrated the role played by BM and CNS microenvironment in developing relapse (Gossai et al., 2017). Several Proteins such as Rho GTPases act on leucocytes and leukemic cells to promote trans-endothelial migration (TEM) that may later trigger organ infiltration (Bogert et al., 2020).

Cortactin, one of sarcoma (Src) kinases, is an actin-binding protein which is expressed in nearly all cells and it is involved in cytoskeletal regulation including cell adhesion and migration (Schnoor et al., 2018). It plays a vital role in invadopodia formation and extra cellular matrix degradation (Kirkbride et al., 2011). Cortactin overexpression is associated with increased metastasis and worse prognosis in different types of solid tumors (Yin et al., 2017) such as head and neck tumors (Timpson et al., 2007), gastric tumors (Wei et al., 2014), and colorectal carcinomas (Jing et al., 2016). Hematopoietic cell-specific lyn substrate-1 (HS1) is a cortactin homologue that is exclusively expressed in hematopoietic cells and has many overlapping functions with cortactin including cell migration (Castro-Ochoa et al., 2016). Recent studies have demonstrated cortactin overexpression to be related to disease progression and bad prognosis in different hematological malignancies such as B-CLL (Gattazzo et al 2014), non-Hodgkin B-cell lymphomas (Pizzi et al., 2019) and B-cell ALL (Velázquez-Avila et al., 2019). Therefore, the aim of the present work is to assess the clinical value of cortactin and HS1expression in B-cell acute lymphoblastic leukemia.

## Materials and Methods

*Patients*

This study was carried out on 74 B-ALL patients (39 male and 35 females, mean age of 38.4 [±12.8] years) attending outpatient clinic of Mansoura University oncology Center. A total number of 76 healthy controls of matched age and gender were included in the study. This study was reviewed and approved by the local Ethics 

*Committees at Mansoura*

Faculty of Medicine, Mansoura University. Written informed consent was taken from patients participating in the study according to the Declaration of Helsinki. 

The diagnosis was based on patients’ history, complete blood counts (CBC), blood smear, bone marrow aspirate smear, LDH level, immunophenotyping and cytogenetic analysis. Patients were classified according to the 2016 WHO criteria. The patient’s characteristics are shown in Table 1.

Bone marrow samples (1 ml EDTA) were collected at two time points; at diagnosis and after induction of chemotherapy. RNA was extracted using QIAamp RNA Blood Mini Kit (Qiagen, Germany), RNA concentration was measured using nanodrop to ensure high RNA quality, and cDNA was synthetized using high capacity cDNA reverse transcription kit (Applied Biosystems, USA).

*Quantitative RT-PCR*

Real time reverse transcription (RT) polymerase chain reactions were performed for both cortactin and *HS1* genes using β2-microglobulin (housekeeping gene) as an internal control. The primer sequences used for q-RT-PCR are as follows: (cortactin forward primer) 5′-GGTGTGCAGACAGACAGACAA-3′, 

(cortactin reverse primer) 

5′-GTCTTTTTGGGATTCATGCAG-3′; (HS1 forward primer) 5′-CCCAAGAGTCCTCTCTATCCTG-3′, (HS1 reverse primer), 5′-GGTGGCAGAGAGGTGTTCAT-3′;

(β2-microglobulin forward primer), 

5′-TCAGGAAATTTGACTTTCCATTC-3′ and 

(β2-microglobulin reverse primer) 

5′-TTCTGGCCTGGAGGCTATC-3′. Analysis was performed using StepOne Real-time Detection Thermal Cycler, USA, and samples were amplified using these conditions: pre-incubation: 95°C, 10 min; amplification: 40 cycles of 95°C, 10 s; 60°C, 30 s; 72°C, 10 s. Relative gene expression was calculated using the ΔΔCt-method.

*Statistical analysis*

Data were analyzed using the Statistical Package of Social Science (SPSS) program for Windows (Standard version 24). Kolmogorov-Smirnov test was used as a test of normality, if the significance level is greater than 0.05, then normality is assumed. Student T Test was used to assess the statistical significance of the difference between two study group means. Mann Whitney Test (U test) was used to assess the statistical significance of the difference of a non-parametric variable between two study groups. Chi-Square test was used to examine the relationship between two qualitative variables. Fisher’s exact test: was used to examine the relationship between two qualitative variables when the expected count is less than 5 in more than 20% of cells. Wilcoxon signed rank sum test was used to assess changes in parameters over time. Logistic regression analysis was used for prediction of risk factors, using generalized linear models. Kaplan–Meier test was used for survival analysis and the statistical significance of differences among curves was determined by Log-Rank test. Cox regression analysis of factors potentially related to survival was performed to identify which independent factors might jointly have a significant influence on survival. A p value is significant if <0.05 at confidence interval 95%.

## Results

*Cortactin and HS1 genes expression in B-ALL versus control*

Cortactin and *HS1* gene expressions were estimated in newly diagnosed 74 B-ALL patients at diagnosis and after induction chemotherapy as well as in B cells from control subjects. The median cortactin expression before and after therapy was 4.01 and 1.19 respectively compared to a median expression of 0.19 in control group (P <0.001 in both). *HS1* expression was measured before and after induction therapy and showed a median of 19.7 and 6.63 respectively compared to 1.16 in control group (P <0.001 in both). Table 2 shows comparison of cortactin and *HS1* expression between studied groups. Cortactin and *HS1 *gene expressions were significantly up regulated in ALL cases before and after treatment when compared to control groups, as well as before when compared to after treatment.

*Impact of the degree of cortactin and HS1 expression at diagnosis on induction remission response*

Stratifying patients according to induction remission response showed that *B-ALL* achieved remission had cortactin gene expression with a median of 4.6 before treatment compared to 1.2 after treatment (P<0.001) and median *HS1* gene expression was 19.7 before treatment compared to 6.6 after treatment (P<0.001). While patients who didn’t achieve complete remission had median cortactin gene expression 2.6 before treatment compared to 0.7 after treatment (P<0.001) and* HS1* gene expression with a median of 14.9 before treatment compared to 5 after treatment (P<0.001). This finding points out that B-ALL patients with high cortactin and *HS1* expression at diagnosis showed low frequency of complete remission after induction chemotherapy as compared to those express high expression (P<0.01 for both) (Table 3).

*Relation between degree of Cortactin and HS1 genes expression levels and different clinicopathological parameters*

High cortactin expression was significantly associated with lower Hb level (median=8, P=002), lower RBCs count (median =3, P= 0.015), lower platelet count (median=23000, P=0.025), higher BM blasts percentage (median 85%, P=0.015), frequency of positive Philadelphia chromosome (n=37.8%, P=0.036), cytogenetic risk (P=0.042), and relapse rate (n=18.9%, P=0.025) (Table 4). High *HS1* expression was only significantly associated with higher relapse rate (n= 21.9%, P=0.018).

*Correlation between Cortactin and HS1 expression in B-ALL*

**Figure 1:**
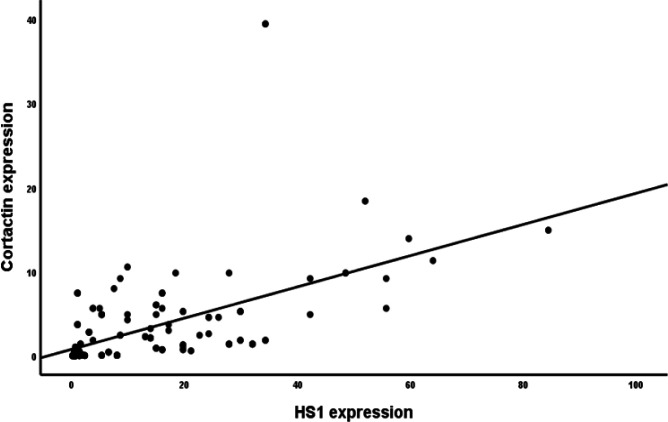
Correlation of Cortactin with *HS1 *Gene Expression in All Studied Cases. A significant positive correlation between the two parameters (R=0.629, p<0.001).

Correlation studies revealed that there is significant positive correlation between cortactin and HS1 expression ((R=0.629, p<0.001) (Figure 1).

*Survival analysis of B-ALL patients*

Using Kaplan Meier curve to assess the Impact of Cortactin and *HS1* gene expression reveals that B-ALL patients with high cortactin expression and *HS1* gene expression had shorter OS as compared to those with low expression (P<0.01 for both) (Figure 2a, 2b). 

*Cox proportional hazard of different clinicopathologic parameters on B-ALL patients outcome*

Cox regression analysis was conducted for assessment the impact of studied parameters on B-ALL patients outcome, using age, gender, blasts percentage, risk Philadelphia chromosome, cortactin and *HS1* genes expression as covariates in univariate analysis. The significant parameters in univariate analysis were evaluated in multivariate analysis which revealed that High LDH, cortactin and *HS1* gene expression were considered as high-risk factors associated with high probability of deaths (P= 0.041, OR= 1.092, CI [1.002-1.046]; and P=0.005, O =1.071, CI [1.013-1,041], respectively) (Table 5).

*Cox proportional hazard of different clinicopathologic parameters on B-ALL patients’ relapse*

Logistic regression analysis was conducted in order to determine the high-risk factors for B-ALL relapse; using age, gender, bone marrow blasts percentage, Philadelphia chromosome, cortactin and *HS1* gene expression as covariates in univariate analysis. The significant parameters in univariate analysis were evaluated in multivariate analysis which revealed that high cortactin and *HS1* gene expression were considered as significant risk predictors of B-ALL relapse (P=0.005, OR=1.428, CI [1.175-1.783]; P=0.003, OR= 1.078, CI [1.025-1.134]; respectively) and high probability of deaths (P= 0.041, OR = 1.092, CI [1.002-1.046]; and P=0.005, OR= 1.071, CI [1.013-1,041]; respectively) (Table 6) (Figure3a, 3b).

**Table 1.** Patients' Characteristics

		ALL (n=74)
TLC (X10^9^/L) Median (IQR)		25.6 (5.8-74.6)
Hb (g/dL) Median( IQR)		8.5 (7.6-9.5)
RBCS (X10^12^/L) Median ( IQR)		3.2 (2.8-3.5)
Platelets (X109/L) Median ( IQR)		35.2 (18-102)
BM blasts (%) Median (IQR)		80 (70-90)
LDH (U/L) Median ( IQR)		612 (429.75-1012.5)
Philadelphia positive N (%)		20 (27.0)
Cytogenetic risk	Standard N (%)	22 (29.7)
	High N (%)	52 (70.3)
Outcome	Remission N ( %)	51 (68.9)
	No remission N ( %)	23 (31.1)
Relapse N (%)		8 (10.8)

N, number; IQR, interquartile range

**Table 2:** Comparison of the Cortactin and *HS1* Genes Expressions in B-ALL Patients before and after Induction Chemotherapy versus Control

		B-cell Control N=76	B-ALL N=74		*P* ^1^	*P* ^2^	*P* ^3^
			At diagnosis Before therapy	After induction chemotherapy			
Cortactin expression	Median	0.19	4.01	1.19	<0.001	<0.001	<0.001
	IQR	0.09-0.54	1.41-6.41	0.38-2			
HS1 expression	Median	1.16	19.7	6.63	<0.001	<0.001	<0.001
	IQR	0.66-2.85	14.93-29.86	5.03-10.24			

*P* ^1^ *,* comparison between ALL before treatment and control; *P*^2^, comparison between ALL before and after treatment; *P*^3^, comparison between ALL after treatment and control.

**Table 3:** Cortactin and *HS1 *Gene Expressions at Diagnosis in B-ALL Patients Aachieved Complete Remission Vs Those Resistant for Induction Chemotherapy

Induction of remission response	Cortactin expression At B-ALL diagnosis Media (IQR)	HS1 expression B-ALL diagnosis Median (IQR)
Yes	14.9 (13-34.3)	2.6 (1.9-9.2)
No	19.7 (16-29.9)	4.6 (1.4-6.1)
P value	<0.001	<0.001

**Table 4:** Comparison of Studied Parameters between Low and High Cortactin Levels at Diagnosis in ALL Cases

	Low cortactin N=37	High cortactin N=37	p
Age (years)	38.7 ±12.5	38.1±11.3	0.888
M±SD			
Males N(%)	16 (43.2)	11(29.7%)	0.227
Females N(%)	21 (56.8%)	26 (70.3%)	
TLC (X10^9^/L)	28.3 (5.8-66.4)	23 (5.3-81)	0.301
Median(IQR)			
Hb (g/dL) Median (IQR)	9 (8-10.2)	8 (7.3-8.95)	0.002
RBCS (X10^12^//L) Median (IQR)	3.3(2.9-3.9)	3(2.6-3.5)	0.015
Platelets (X10^9^/L) Median (IQR)	46(19.9-128.6)	23(14.3-76)	0.025
BM blasts (%) Median (IQR)	80 (65.5-80)	85(70-90)	0.015
LDH (U/L) Median (IQR)	607 (459.5-930)	789 (404-1100.5)	0.996
Philadelphia positive N (%)	6 (16.2%)	14( 37.8%)	0.036
Standard cytogenetic risk N(%)	30 (81.1%)	22 (59.5%)	0.042
High cytogenetic risk N(%)	7 (18.9%)	15 (40.5%)	
Remission N (%)	24 (64.9%)	10 (27.0%)	0.0451
Non remission N (%)	13 (35.1%)	27 (73.0%)	
Relapse N (%)	1 (2.7%)	7 (18.9%)	0.025
*HS1* expression Median (IQR	19.7 (14.4-27.9)	24.3 (14.9-42.2)	0.192

**Figure 2a:**
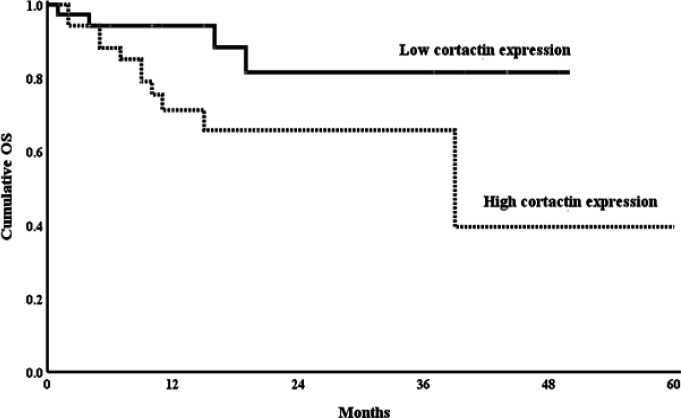
Kaplan-Meier Plot of the OS Probability in the High and Low Cortactin Groups

**Table 5:** Cox Regression Analysis for Prediction of OS in B-ALL Patients

	Univariable				Multivariable			
	p	HR	95% CI		p	HR	95% CI	
Age	0.02	1.038	1.006	1.071				
Gender	0.211	0.448	0.127	1.578				
BM blasts%	0.982	0.998	0.956	1.044				
LDH (U/L)	0.014	1.012	1.003	1.031	0.005	1.022	1.004	1.051
Cytogenetic risk	0.168	2.848	0.643	12.621				
Philadelphia	0.936	1.048	0.336	3.27				
Cortactin expression	0.009	1.047	1.012	1.083	0.041	1.046	1.002	1.092
*HS1 *expression	<0.001	1.051	1.024	1.078	0.005	1.041	1.013	1.071

HR, hazard ratio; CI, confidence interval

**Figure 2b:**
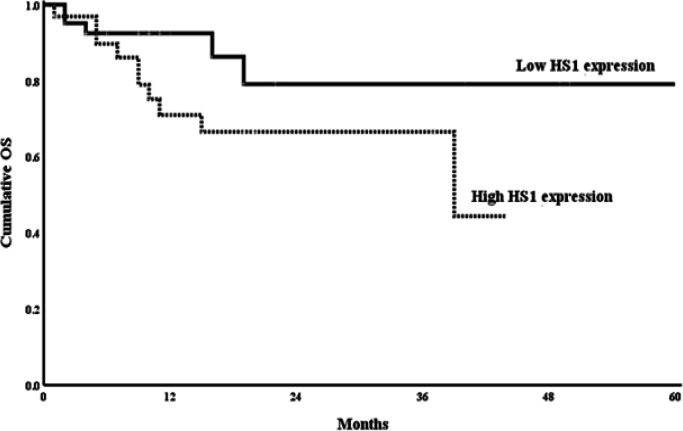
Kaplan-Meier Plot of the OS Probability in the High and Low HS1 Groups

**Figure 3a:**
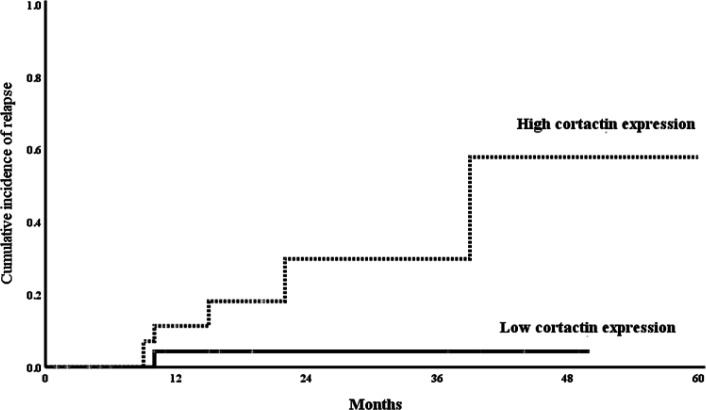
Comparison of the Cumulative Incidence of Rrelapse (CIR) Curves between the Low and High Cortactin Gene Expressions Groups

**Table 6:** Cox Regression Analysis for Prediction or Relapse in ALL Cases

		No relapse	Relapse	Univariable			Multivariable		
		N=43	N=8	p	OR	95% CI	p	OR	95% CI
Age	Mean ±SD	33.6 ±11.1	36.4 ±10.2	0.166	1.023	0.991- 1.056			
Males	N	26	7		1				
	%	60.50%	87.50%						
Females	N	17	1	0.142	0.452	0.157-			
	%	39.50%	12.50%			1.304			
BM blasts%	Median	80	80	0.514	0.988	0.952-1.025			
	IQR	70-90	70-88						
LDH (U/L)	Median	607	402	0.339	0.999	0.998-1.001			
	IQR	435-930	176-977						
Standard cytogenetic risk	N	17	2		1				
	%	39.50%	25.00%						
High cytogenetic risk	N	26	6	0.431	1.44	0.580-3.575			
	%	60.50%	75.00%						
Philadelphia negative	N	34	5		1				
	%	79.10%	62.50%						
Philadelphia positive	N	9	3	0.326	1.585	0.632-3.974			
	%	20.90%	37.50%						
Cortactin expression	Median	3	10.3	0.012	1.065	1.014-1.119	0.005	1.428	1.175-1.783
	IQR	1.4-5.3	5.4-17.3						
*HS1* expression	Median	19.7	47.1	0.001	1.088	1.035-1.143	0.003	1.078	1.025-1.134
	IQR	16-27.9	25.5-58.7						

OR, odds ratio; CI, confidence interval.

**Figure 3b:**
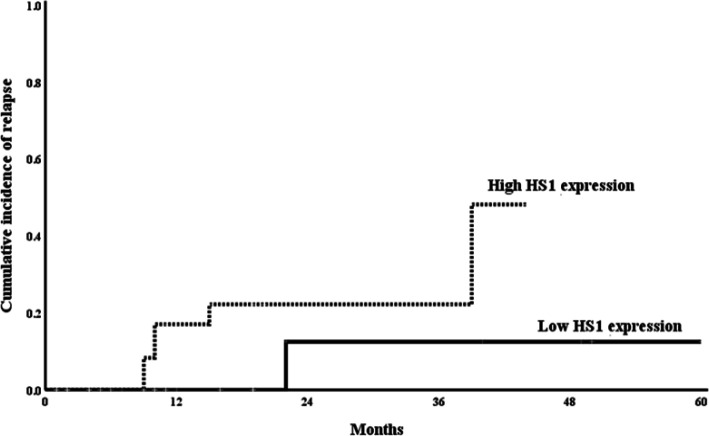
Comparison of the Cumulative Incidence of Relapse (CIR) Curves between the Low and High *HS1* Gene Expression

## Discussion

Cortactin the actin-nucleation-promoting factor is virtually expressed on all cell types and is important for cell functions, such as adhesion and migration (Kirkbride et al., 2011). Cortactin has emerged not only as a crucial regulator of actin cytoskeletal dynamics, but also as a key player in aggressive cancers (McGrath and Koleske., 2012). 

In the current study high expression of cortactin as well as its homologue HS1 was detected in B-ALL patients at diagnosis and significantly reduced after induction chemotherapy as compared to controls. These findings were in agreement with that reported by Velázquez-Avila et al., (2019), who reported higher expression of both cortactin and HS1 among B-ALL patients. Also; previous report stated that cortactin is strongly overexpressed in leukemic cell lines and primary patient-derived leukemic cells (Yin et al., 2017).

In our study, high cortactin gene expression was significantly associated with lower Hb level, lower RBCs count, lower platelet count and higher BM blasts. Also, high cortactin expression was significantly associated with higher cytogenetic risk as well as high relapse rate. These findings point out for the role of cortactin in blast cells egress from bone marrow into peripheral blood and peripheral tissues which increase tumor burden. Cortactin overexpression is associated with increased metastasis formation and worse outcome in different types of solid tumors, thus highlighting a critical role of cortactin in cancer progression. Mechanistically, this is due to increased invadopodia formation and matrix metalloproteinase secretion (Gattazzo et al., 2012; Yin et al., 2017). Additive report indicated that overexpression of cortactin has been reported in several solid tumors, and increased expression of *CTTN*, the gene encoding for cortactin, has been associated with aggressive, poor prognosis disease, the investigators noted (Pizzi et al., 2019).

In the present study higher cortactin and* HS1* genes expressions were also associated with shorter OS and high frequency of B-ALL relapse. These findings were in parallel to the findings reported by Velázquez-Avila et al., 2109). Moreover; high cortactin expression was reported in B-cell chronic lymphocytic leukemia, and is associated with poor prognosis and increased chemotaxis; and is correlated with treatment failure and relapse (McGrath and Koleske 2012). In addition; high cortactin expression has been proposed as a diagnostic marker for non-Hodgkin B-cell lymphomas (Pizzi et al., 2019). Also; Gattazzo et al., (2012) suggested that cortactin is involved in aggressiveness and spreading of B-CLL cells and that Lyn-cortactin axis could represent an alternative target for the development of new therapeutic strategies.

Using Cox regression analysis model for prediction of OS; In univariate analysis the parameters showed higher risk include high LDH level, high cortactin and* HS1 *gene expression; while in multivariate analysis high cortactin and* HS1 *gene expressions were considered as risk predictors of relapse in uni- and multivariable analyses. 

In conclusion, high cortactin and/or HS1 expression is associated with a worse outcome for B-ALL patients. These findings suggest that determining cortactin levels at B-ALL diagnosis could be used in risk stratification and optimizing their treatments. Moreover; cortactin might be a potential pharmacologic target to control the metastasis or relapse of B-ALL.

## Author Contribution Statement

Study conception and design: Salah Aref, data collection : Tarek Abou Zeid analysis and interpretation of results: Mohamed Sabry and Nada Khaled; draft manuscript preparation: Mohamed Al Agdar and Ahmed Ramez. All authors reviewed the results and approved the final version of the manuscript.
